# Femoral neck fractures

**DOI:** 10.4103/0019-5413.38573

**Published:** 2008

**Authors:** Hardas S Sandhu, Mandeep S Dhillon, Anil K Jain

**Affiliations:** Kitty Cottage, 183 Circular Road, Amritsar, India; 1 PGI Chandigarh, India; 2 University College of Medical Sciences and Indian Journal of Orthopaedics, Delhi - 110 095, India. E-mail: dranilkjain@gmail.com

The femoral neck fracture continues to be unsolved fractures and the guidelines for management are still evolving. It is a common skeletal injury, occurring with minor trauma in the osteoporotic bone of elderly patients. In younger patients (<50 years), it occurs due to high velocity trauma and may be a part of poly-trauma, with multiple fractures including that of ipsilateral femur. Somewhat less frequently, it is also seen in children.

The management of this fracture has evolved significantly. The management started with closed reduction and immobilization in POP hip spica in abduction and internal rotation (Whitman Abduction plaster) in the early part of 20^th^ century. High incidence of non-union, bed sores and respiratory complications led to exploration of methods of internal fixation. The introduction of Smith Peterson nail brought a new hope of solving the problem, but high failure and complication rates disappointed many surgeons. Further improvement in implant designs brought in newer devices (nail plate assemblies) like Smith Peterson nail plate and McLaughlin nail plate; these also did not withstand the test of time. The modern concepts of fixation under compression led to the use of cancellous partially threaded screws and placement over preliminary wires led to the development of cannulated variety of screws, which are now the standard of care in adults. Smooth pins (Moore or Knowles pins) are still the choice for children.

The presentation at different ages poses different problems related to the management. The issues are fixation failure in osteopenic bone of the elderly, marked displacement of fragments posterior comminution and disruption of blood supply in young adults, and a higher incidence of avascular necrosis (AVN) in young adults and children. In children, the growth plate needs to be protected by using implants which would minimize damage without compromising on the stability of reduction and internal fixation. Femoral neck fracture is unique because of its anatomical and biomechanical consideration. The blood supply to the femoral head comes from three main sources, i.e., medial femoral circumflex artery, lateral femoral circumflex artery and obturator artery through intracapsular terminal branches which run parallel to the neck. Any femoral neck fracture disrupts the terminal blood vessels producing AVN. The fracture is intra-articular and this exposes the fracture surfaces to synovial fluid and its enzymes. Depending on the fracture configuration and the action of various groups of muscles acting on the hip, it is subjected to a very high degree of shearing strain. Hence, accurate reduction and internal fixation are mandatory requirements to expect fracture healing.

In patients presenting early, the decision to operate in the emergency scenario or not is also debatable although no difference in the AVN rates has been reported in patients operated by closed or open reduction or if they are operated early or with delays. The outcome is further complicated if these cases reach late by a few days to few weeks, after getting treatment from “Quacks” in the form of massage and manipulations. They may present after treatment which may have been non-operative, the fracture poorly reduced and inadequately stabilized. Surgeons often see patients with smoothened fracture surfaces and increased fracture gap due to femoral neck resorption and a reduced size of the proximal fragment. Even the features of AVN may be manifest on primary radiological examination.

The issues to resolve at initial presentation in fresh fractures are traction or no traction while waiting for surgery, necessity for capsular decompression, closed or open reduction, acceptability of reduction, internal fixation, valgization or no valgization, need for primary valgization in fresh fractures or only in cases with late presentation. Late presentation at 3 weeks, 3 months, 6 months or more have different outcomes; the guidelines to evaluate and manage such cases are still to be established. In the Indian scenario, the focus is always on femoral head preservation. In the elderly, however, the options in managing femoral neck fractures seem to be more in favour of arthroplasty and the procedure may vary from cemented to uncemented fixation, and Bipolar to total joint replacement.

The management of femoral neck fractures in young adults is the focus of four articles in this symposium. The review article by Drs. Thuan Ly and Swiontkowski states that the key factors in treating femoral neck fractures seem to be early diagnosis, early surgery, anatomic reduction, capsular decompression and stable internal fixation. They have advocated open reduction whenever even a semblance of doubt exists about the accuracy of reduction, emphasizing reduction accuracy as the key to better union rates.

Even after achieving anatomical reduction and stable fixation, there still remains some doubt about achieving union. Failure of fixation and AVN are considered to be responsible for this. Even after the fracture is united, AVN and delayed segmental collapse of femoral head can occur, predisposing the joint to early osteoarthrosis. Many procedures for aiding fracture union and reducing AVN incidence have been advocated; the options range from osteotomies to converting the shearing stresses into compressive ones and aiding union and revascularization by muscle pedicle graft or free fibular grafts. In the present symposium, we have two manuscripts dealing with attempts to primarily reduce these complications in displaced femoral neck fractures. The first advocates closed reduction of fresh fractures and internal fixation, followed by Quadratus femoris muscle pedicle bone graft; the other reports on femoral neck fractures older than 3 weeks, where open reduction and Meyer's muscle pedicle graft were performed. Dr. MP Singh and co-authors report on 55 young adults with displaced femoral neck fractures less than 3 weeks old, managed by osteosynthesis and primary valgus intertrochanteric osteotomy using contoured broad dynamic compression plate, achieving 92.7% union rates.

Osteotomies for the management of non-union or neglected cases of femoral neck fracture have been reported as a viable option to aid union. McMurrays osteotomy is no longer in vogue, but valgus osteotomies that convert the shear forces to compressive forces at the fracture site, increasing implant stability and allowing faster healing, are increasingly being used. Drs. Raaymakers and Marti in their article state, “In the competition between revascularization and collapse, often revascularization will be the winner". They operated 66 young patients of non-union of femoral neck fracture over 22 years by a Pauwel's valgization osteotomy and obtained 88% femoral neck union and overall 62% good results. They have recommended an attempt to save the femoral head even with collapse, as it could delay arthroplasty. Total hip arthroplasty is considerably postponed and better conditions for hip replacement can be achieved by the development of sclerotic bone in the subchondral areas of the acetabulum and femoral head. Magu reported comparable results in two groups, albeit non-randomized, of elderly patients with THR and valgus osteotomy. Arthroplasty has the advantage of early patient mobilization, but the cost factor and specific activity limitations are significant issues in patients who have to squat or sit on the ground for activities of daily living. It is definitely not a practical proposition for labourers and heavy manual workers in the younger age groups.

Femoral neck fracture in the elderly poses a problem of poor bone quality and co-morbid medical conditions. Hagino *et al*. evaluated the prognosis among different age groups in elderly patients aged 65 years and above treated for hip fractures. Replacement arthroplasty, either hemiarthroplasty (Austin Moore, Thompson's or Bipolar prosthesis) or total hip replacement was found to be ideally suited for the elderly population as a primary procedure to tackle the problem of fixation failure, non-union and AVN. In this symposium also, two articles are focussed on prosthetic replacement in femoral neck fractures in the elderly. THA is advocated in cases where life expectancy is significant and when acetabular disease is present. For older people, bipolar arthroplasty may be a better option. Specific indications exist for cemented and cementless replacements. Yusuf, in his article, stressed the advantage of uncemented fixation of hemi or total hip arthroplasty in femoral neck fractures in elderly patients with co-morbidities.

This symposium is an attempt to dwell on some of these issues and discuss the future directions in the management of femoral neck fractures. Efforts will continue to be made to achieve better results. Internal fixation of the fracture in combination with the use of osteo-inductive materials may be a new lead to further improvement of the results.

